# Hypertrophic cardiomyopathy and the myosin mesa: viewing an old disease in a new light

**DOI:** 10.1007/s12551-017-0274-6

**Published:** 2017-07-17

**Authors:** Darshan V. Trivedi, Arjun S. Adhikari, Saswata S. Sarkar, Kathleen M. Ruppel, James A. Spudich

**Affiliations:** 1 Department of Biochemistry, Stanford University School of Medicine, Stanford, CA 94305 USA; 2 Department of Pediatrics (Cardiology), Stanford University School of Medicine, Stanford, CA 94305 USA

**Keywords:** Hypertrophic cardiomyopathy, Dilated cardiomyopathy, Interacting-heads motif, Myosin sequestered state, Myosin mesa, Myosin binding protein C

## Abstract

The sarcomere is an exquisitely designed apparatus that is capable of generating force, which in the case of the heart results in the pumping of blood throughout the body. At the molecular level, an ATP-dependent interaction of myosin with actin drives the contraction and force generation of the sarcomere. Over the past six decades, work on muscle has yielded tremendous insights into the workings of the sarcomeric system. We now stand on the cusp where the acquired knowledge of how the sarcomere contracts and how that contraction is regulated can be extended to an understanding of the molecular mechanisms of sarcomeric diseases, such as hypertrophic cardiomyopathy (HCM). In this review we present a picture that combines current knowledge of the myosin mesa, the sequestered state of myosin heads on the thick filament, known as the interacting-heads motif (IHM), their possible interaction with myosin binding protein C (MyBP-C) and how these interactions can be abrogated leading to hyper-contractility, a key clinical manifestation of HCM. We discuss the structural and functional basis of the IHM state of the myosin heads and identify HCM-causing mutations that can directly impact the equilibrium between the ‘on state’ of the myosin heads (the open state) and the IHM ‘off state’. We also hypothesize a role of MyBP-C in helping to maintain myosin heads in the IHM state on the thick filament, allowing release in a graded manner upon adrenergic stimulation. By viewing clinical hyper-contractility as the result of the destabilization of the IHM state, our aim is to view an old disease in a new light.

## Introduction

**Fig. 1:**
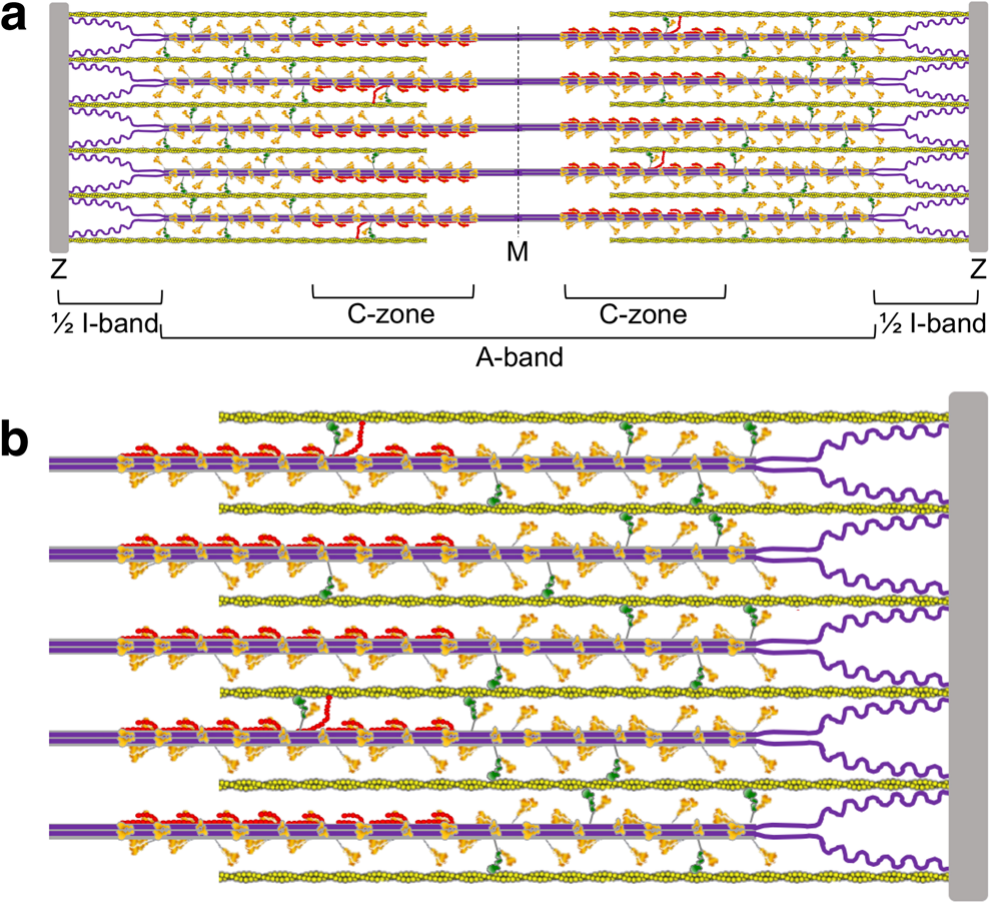
Schematic description of a cardiac sarcomere drawn to scale. **a** A sarcomere is depicted at its resting length just beginning its contraction. The sarcomere is 2 μm long, and the myosin bipolar thick filaments are 1.6 μm long and constitute the A-band. The actin filaments are 0.8 μm long. The zone containing actin with no myosin overlap is shown as the ½ I-band; the other half of the rightmost I-band is just to the right of the Z-disc in the neighboring sarcomere (not shown). Titin (*purple*) is attached to the Z-disc and extends to the M-line, where it overlaps with titin from the other half of the sarcomere. Myosin binding protein C (MyBP-C; *red*) is located in the C-zone. Most MyBP-C molecules shown here are sequestering folded-back myosin heads; four cases are shown where the MyBP-C has dissociated from the myosin heads, freeing them to enter the chemomechanical cycle and allowing the N-terminal domain of MyBP-C to interact with actin. **b** The total number of myosin molecules in a half-sarcomere directed toward one actin filament in this schematic model is 48. In this schematic model, a maximum of eight of these 48 molecules (17%) are sequestered by MyBP-C (*red*) holding them in a folded-back state not available for interaction with actin. Of the remaining 40 heads that are not bound to MyBP-C, we show 50% (the percentage regulated by regulatory light chain phosphorylation) of them in a folded-back interacting-heads motif state by head interactions with their own S2 tails and other interactions that prevent them from interacting with actin. This leaves 20 myosin molecules free to interact with actin. If the duty ratio under some load is approximately 0.2, then only approximately four myosin molecules are interacting with a given actin filament at any moment during systolic contraction

The study of muscle biology has been historically filled with dogmas that were difficult to negate simply due to long-standing beliefs. The sliding filament theory of muscle contraction introduced in the early 1950s (Huxley 1953; Huxley and Hanson 1954; Huxley and Niedergerke 1954) was the first step in the process of overturning the long-standing view that muscle contraction involved the coiling or folding of fibrous structural elements that consisted of continuous actin and myosin proteins. Even after these early papers in which the sliding of filaments was proposed, many investigators continued to argue for the old view of muscle contraction—the notion of coiling or folding of filaments was simply too attractive and obvious to be wrong (Rall 2014). The turning point for full acceptance of the sliding filament theory was the publication of remarkable thin section electron micrographs by Hugh Huxley (Huxley 1957). These data were so convincing as to leave no doubt that the actin and myosin filaments were in fact not continuous (Fig. 1) and showed different degrees of overlap in resting and relaxed states (Huxley 1957).

**Fig. 2:**
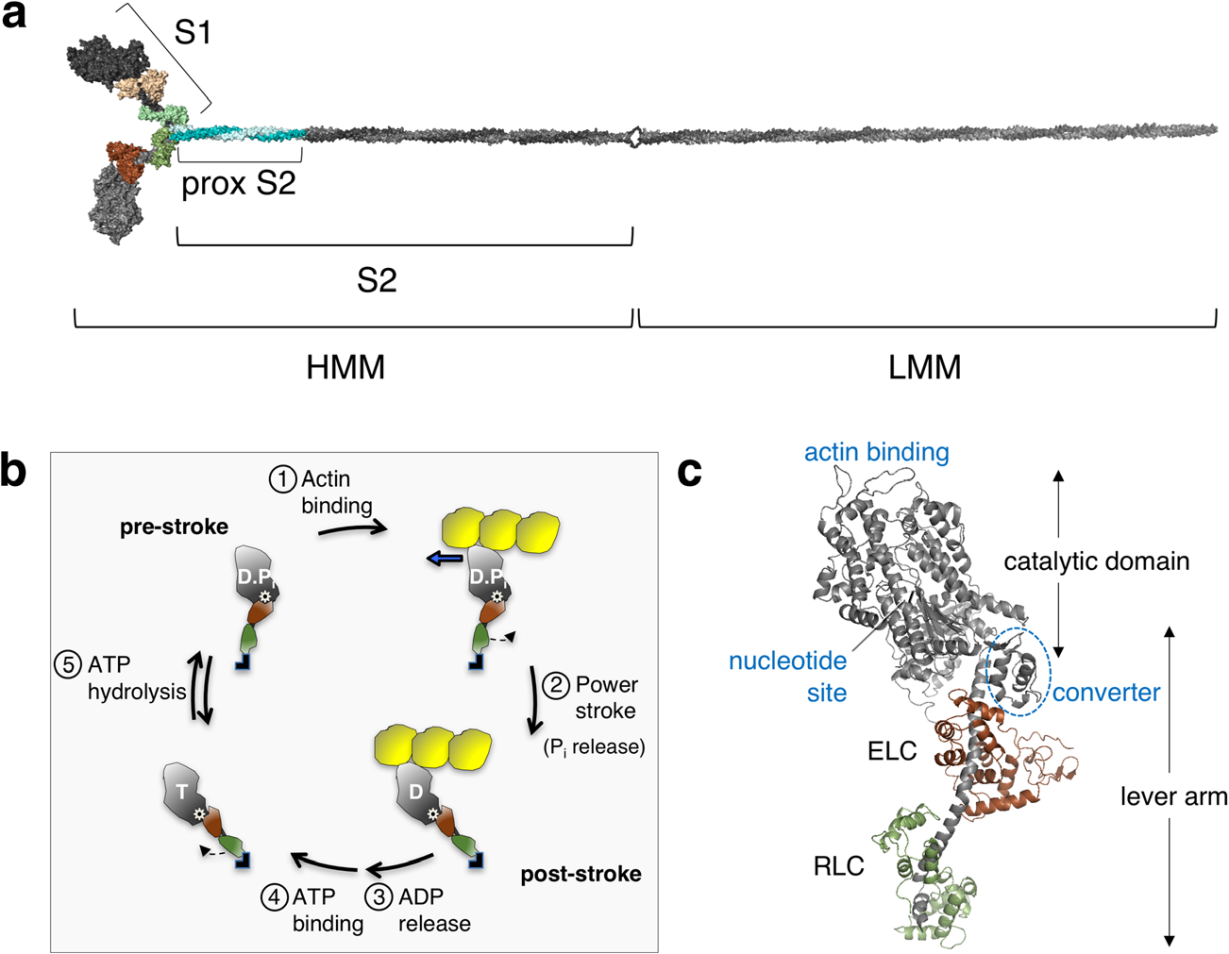
Human β-cardiac myosin structural models and the chemomechanical cycle. **a** PyMOL-rendered homology model of the full-length human β-cardiac myosin molecule showing the subfragment 1 (*S1*), proximal subfragment 2 (*prox S2*), subfragment 2 (*S2*) and heavy and light meromyosin (*HMM* and *LMM*, respectively) domains. The templates used to model the post-stroke structure were obtained from the human β-cardiac myosin motor domain solved by Winkelmann et al. (2015), supplemented with the rigor structure from the squid myosin motor domain (Yang et al. 2007), as described in Nag et al. (2017). The S2 region is a long coiled-coil structure; hence we used the template from the Myosinome database (Syamaladevi et al. 2012). Modeling was done using the MODELLER package. **b** Simplified chemomechanical cycle of the interaction of myosin heads with actin. Steps of the chemomechanical cycle are: *1* The pre-stroke S1 with bound ADP (*D*) and inorganic phosphate (*P*_*i*_) binds to actin (*yellow*); *2* while bound to actin, the lever arm swings to the right about a fulcrum point (*black dot on white star*) to the post-stroke position, moving the actin filament to the left (*bold blue arrow*) with respect to the myosin thick filament; *3* ADP release frees the active site for binding of ATP (*T*); *4* ATP binding weakens the interaction of the S1 to actin; *5* ATP hydrolysis locks the head into the pre-stroke state. **c** Homology-modeled human β-cardiac myosin S1 in its pre-stroke state showing various important domains of this subfragment of myosin. *ELC* essential light chain, *RLC* regulatory light chain

A decade later Hugh Huxley proposed the swinging cross-bridge model of muscle contraction (Huxley 1969), which was immediately accepted as the most plausible of hypotheses and soon appeared in the early cell biology textbooks as the model of how muscle works. According to this model, each myosin head, known as subfragment 1 (S1) of myosin (Fig. 2), binds to an actin filament and undergoes a power stroke of approximately 10 nm, thereby generating the force and velocity of muscle contraction. This attractive hypothesis, however, gradually fell out of favor between 1969 and the early 1980s due to contrary evidence from biophysical studies (Cooke 1986, 2004) and suggestions that the step size taken by myosin per ATP utilized may be >60 nm (Harada et al. 1990; Yanagida and Iwane 2000), which is a step size too large to be consistent with the swinging cross-bridge model. Again, it appeared that dogma may be overturned. However, the advent of in vitro motility assays for myosin-based movement of actin filaments with purified proteins (Kron and Spudich 1986; Spudich et al. 1985), the demonstration that the myosin head S1 is sufficient to induce sliding movement of (Toyoshima et al. 1987) and tension on (Kishino and Yanagida 1988) actin filaments, the determination in 1993 (Rayment et al. 1993) and beyond (Geeves and Holmes 1999; Rall 2014) of crystal structures of S1 revealing a light chain-bound putative lever arm to amplify movements during contraction (Fig. 2), the demonstration in 1994 of myosin step sizes of approximately 10 nm by single molecule methods (Finer et al. 1994), and a host of studies involving myosin V and myosin VI (for reviews, see Dantzig et al. 2006; Holmes et al. 2004; Olivares and De La Cruz 2005; Sellers and Veigel 2006; Spudich 2012; Spudich and Sivaramakrishnan 2010; Sweeney and Houdusse 2004, 2007; Trybus 2008; Vale 2003) all explained the earlier seemingly contrary evidence and proved to be consistent with the swinging cross-bridge model (now the “swinging lever arm” model) of muscle contraction that is currently popular (Fig. 2b).

In 2017 we face another commonly held belief that is taking a turn—this time regarding our understanding of the molecular basis for clinical hyper-contractility caused by hypertrophic cardiomyopathy (HCM) mutations in human β-cardiac myosin, where approximately 40% of all known HCM mutations lie (Buvoli et al. 2008; Colegrave and Peckham 2014; Harris et al. 2011; Seidman and Seidman 2001; Walsh et al. 2010). Based on studies using mouse α-cardiac myosin as a model system, significant increases in intrinsic force of the HCM mutant myosins, and increases in their ATPase activity and their velocity as measured by in vitro motility systems, seemed to easily explain the hyper-contractility seen clinically, and this view has dominated thinking in the field (Moore et al. 2012; Sivaramakrishnan et al. 2009). As explained below, however, recent results using purified human β-cardiac myosin do not fit this paradigm. Our research group and those of many others were not paying sufficient attention to another possible pivotal factor involved in HCM, namely the number of myosin heads in the sarcomere that are functionally accessible for interaction with actin (N_a_). As discussed below, the importance of the physiological regulation of N_a_ in striated muscle has been discussed in studies by Padron and colleagues (Alamo et al. 2008; Brito et al. 2011) and others; so this concept is not new. As also described below, several investigators have pointed out the positions of several HCM and dilated cardiomyopathy (DCM) mutations on myosin and the roles they may play in altering N_a_. We have extended these ideas and have hypothesized (Adhikari et al. 2016; Kawana et al. 2017; Nag et al. 2015, 2017; Spudich 2015; Spudich et al. 2016) that increases in N_a_ may be responsible in the majority of cases for the hyper-contractility caused by HCM mutations, both in myosin and in myosin binding protein C (MyBP-C), and this is a major subject of this review.

Muscle myosin is a hexamer consisting of two myosin heavy chains and two sets of light chains, i.e. the essential light chain (ELC) and the regulatory light chain (RLC) (Fig. 2a, c). The entire myosin molecule when proteolytically digested by chymotrypsin yields two fragments by a cut in the tail region, giving heavy meromyosin (HMM) and light meromyosin (LMM) fragments (Fig. 2a). The HMM fragment contains the two globular heads and approximately 40% of the coiled-coil tail. HMM can be further digested by papain to yield S1 and subfragment 2 (S2). S1 houses the ATP and actin binding sites followed by an α-helix that binds the ELC and the RLC. The transition between the pre-stroke and post-stroke states involves a swing of the converter domain (Fig. 2c) through an angle of approximately 70°, about a fulcrum point marked by the white star with black center in Fig. 2b. The converter swing distance is amplified by the light chain binding domain lever arm (Fig. 2b, c). The RLC can be phosphorylated, which activates the contraction of the sarcomere (Kamm and Stull 2011; Vandenboom 2016). This light chain phosphorylation turns out to be an important part of the story that we review here and is the subject of discussion further in this review.

The myosin thick filament is formed by the interaction of the LMM regions of myosin molecules packed together to form the cylindrical backbone of the bipolar thick filament. Typically, the bipolar thick filament of the human cardiac sarcomere is 1.6 μm long and 20 nm wide (Fig. 1). Myosin heads are arranged on the thick filament backbone in a helical or quasi-helical fashion. There is a 14.3-nm spacing between two adjacent myosin molecules on the filament, with a true repeat of 42.9 nm.

**Fig. 3:**
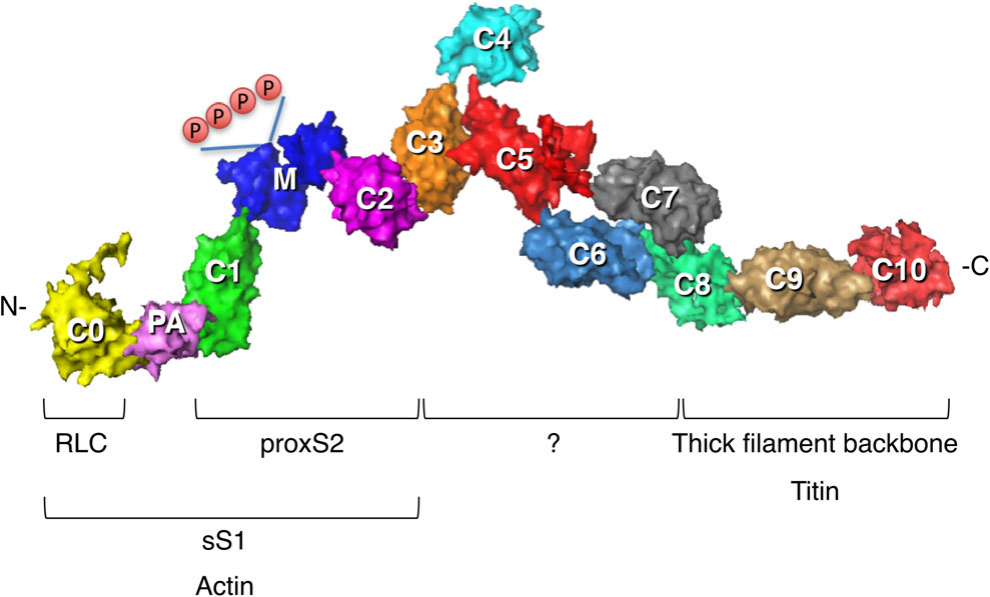
Homology-modeled structure of human cardiac MyBP-C. Modeling was as described in Nag et al. (2017). Surface rendition of full-length human cardiac MyBP-C homology-modeled from known structures of the C0, C1, C2, C3, C5 and M2 domains, which were obtained individually using structural homologues from their respective Protein Data Bank (PDB) files. The other domain structures (C4, C6, C7, C8, C9, C10, M1, PA loop) were modeled independently using Ab Initio (Ab Initio Software, Lexington, MA) and template-based prediction methods. The C0–C10 domains were connected C-terminus to N-terminus using PyMOL software (Schrödinger, LLC, New York, NY) to obtain the image shown. There are four serine phosphorylation sites (*P*) on the M domain (*blue*) that regulate MyBP-C function (Jia et al. 2010). Interactions with other proteins derived from experimental evidence are shown

The thick filament also consists of MyBP-C (Fig. 3), which is found in the C-zone of the thick filaments (Fig. 1). The ratio of MyBP-C to myosin is approximately 1:6 in the sarcomere (1:3 in the C-zone) (Craig and Offer 1976). MyBP-C, identified in 1971 (Starr and Offer 1971), continues to be studied in detail, and interactions with actin (for review, see Craig et al. 2014; van Dijk et al. 2014), the myosin RLC (Ratti et al. 2011), S1 lacking the RLC (Nag et al. 2017), and the proximal part of the S2 tail (proximal S2) (Gruen and Gautel 1999) are thought to play important roles (discussed below). There is much to be learned, however, about its regulatory roles in contraction. The roles of the C3–C7 region of the molecule, for example, have not been studied.

The major protein in the thin filament is actin, with which myosin interacts to generate contractile force. The other components of the thin filament are the Ca^2+^-regulatory proteins tropomyosin and troponin (troponin C, troponin I and troponin T). The thin filament Ca^2+^-regulation system involving tropomyosin–troponin has been thoroughly reviewed by previous authors (Brown and Cohen 2005; Gordon et al. 2000; Lehman 2016; Moore et al. 2016; Tobacman 1996; Zot and Potter 1987) and will not be discussed further here.

Another important protein of the sarcomere is titin, the main subject of the chapters in this Special Issue. Titin is a giant protein that spans half the sarcomere, with its N-terminus embedded in the Z-disk and its C-terminal region interacting with the myosin thick filament (Freiburg and Gautel 1996; Houmeida et al. 1995; Obermann et al. 1997) (Fig. 1). Titin modulates the stiffness of the muscle, with its extensible I-band region acting as a molecular spring that develops passive force when the sarcomere is stretched during diastolic filling. Other regions of the protein have been shown to participate in force-dependent signaling (Linke 2008). HCM mutations are very rare in titin, limited primarily to missense mutations that increase titin’s interactions with binding partners, such as alpha-actinin and four and a half LIM protein 2 (FHL2) (Matsumoto et al. 2005; Satoh et al. 1999). In contrast, 30% of patients with DCM carry mutations in the titin gene (Herman et al. 2012), mostly localized to the A-band region (Fig. 1a). A fundamental difference between HCM and DCM is that HCM results in clinical hyper-contractility while DCM results in hypo-contractility. Mutations that cause defects in the contractile apparatus are intellectually easy to understand—if you do anything to break the system, you will observe hypo-contractility. Hyper-contractility caused by HCM mutations, however, is intellectually a less obvious circumstance—these mutations cause the contractile apparatus to produce higher power output than normal! How this may be explained mechanistically is one focus of this review. Here we emphasize regulation of the thick filament system in striated muscle, primarily cardiac muscle, with a focus on a conformation of myosin that has its S1 heads folded back on their own S2 tail and interacting with each other. This state has been referred to as the interacting-heads motif (IHM) (Alamo et al. 2008). The heads in the IHM are said to be in an ‘off state’, since they are not accessible for interaction with actin. We argue that a majority of myosin HCM mutations, and possibly most MyBP-C mutations as well, likely weaken this IHM state, resulting in more functionally accessible myosin heads for actin interaction, thus causing the hyper-contractility observed for HCM clinically. Rather than simply describe the current status of thinking in the field, we make an effort to clarify what is known, what is speculative and what needs to be done going forward.

## Power output by the sarcomere is the ensemble force of the system times the velocity of contraction

Power is the product of the force and velocity of the contraction, and the force–velocity curve is a fundamental functional aspect of cardiac muscle function. In cardiac muscle, the force axis is related to the load imposed by the systemic circulation against which the ventricle contracts. The ensemble force (F_ensemble_) produced by the muscle must overcome that load in order to pump blood out of the left ventricle during systole. The unloaded velocity (v) is related to the expression v = d/t_s_, where d is the displacement caused by the myosin powerstroke and t_s_ is the strongly bound state time (Howard 2001; Spudich 1990, 1994). F_ensemble_ can in turn be explained by the following relationship:

1 $$ {\mathrm{F}}_{\mathrm{ensemble}}={\mathrm{F}}_{\mathrm{intrinsic}}\cdot {\mathrm{N}}_{\mathrm{a}}\cdot \mathrm{duty}\ \mathrm{ratio} $$

Each head of myosin is an individual force generator and can produce its own intrinsic force (F_intrinsic_). N_a_ is the number of myosin heads on the thick filament that are ‘functionally accessible’ for a fruitful interaction with actin. The duty ratio is a term that reflects the fraction of those accessible myosin heads that are bound to actin in a strongly bound, force-generating state at any moment during contraction. It is the ratio of the strongly bound state time (t_s_) divided by the total cycle time (t_c_) of the actin-activated myosin chemomechanical cycle (Fig. 2b). For cardiac muscle under some load, the duty ratio is approximately 0.2 (depicted in Fig. 1). As discussed below and depicted in Fig. 1, N_a_ is not all of the myosin heads that are physically present in the sarcomere, but may be as low as only 50% of those heads (Brito et al. 2011). It’s the proposed approximately 50% of heads that are not functionally accessible for interaction with actin that offers exciting new revelations in muscle research—and is the focus of this review.

## The myosin mesa and a possible unifying hypothesis for the clinical hyper-contractility caused by HCM mutations

Hundreds of missense mutations in both β-cardiac myosin and MyBP-C are responsible for the devastating inherited disease hypertrophic cardiomyopathy. As already described, approximately 40% of the mutations leading to HCM are found in β-cardiac myosin itself and another 40% in MyBP-C. Those numbers, derived from human genetics studies alone, tell us that these two proteins in particular are somehow speaking to each other to regulate contractility and that mutations in either result in hyper-contractility clinically.

Genetically-based HCM, an autosomal dominant inherited disease (Geisterfer-Lowrance et al. 1990; Seidman and Seidman 2000, 2001), is clinically characterized by hypertrophy of the ventricular walls with a resultant decrease in ventricular chamber size. Systolic performance of the heart is preserved or even increased, but relaxation capacity is diminished. HCM affects more than one in 500 individuals (Harvey and Leinwand 2011; Maron 2010; Maron et al. 1995; Semsarian et al. 2015) and is the most common cause of sudden cardiac death in young adults (Harvey and Leinwand 2011). Current treatment for HCM is limited to symptomatic relief and includes heart muscle reduction surgery (myectomy), defibrillator placement and even heart transplant in the most refractory cases. There is an urgent need for new therapeutic approaches to the disease, but first we need to fully understand its underlying molecular basis. To do so, it is important to reconstitute the important elements of the sarcomere from purified proteins using human cardiac isoforms.

The importance of using purified, homogeneous human cardiac isoforms is emphasized by the variable results obtained using model systems and human biopsy samples. Many studies, largely using the mouse ventricular α-cardiac myosin as a model, have contributed to our current understanding of the effects of some HCM mutations on various functions of the purified actin–myosin complex, usually its ATPase activity or its velocity in the in vitro motility assay (for review, see Moore et al. 2012; Sivaramakrishnan et al. 2009). Although several cardiomyopathy mutations in β-cardiac myosin have been studied over the past 15 years, there has been significant disagreement expressed in the literature regarding the effects of these mutations on the biochemical and biophysical properties of cardiac myosins (reviewed in Moore et al. 2012).

The first HCM-causing mutation in β-cardiac myosin to be identified was R403Q (Geisterfer-Lowrance et al. 1990). Initial studies on this mutant isolated from human soleus muscle biopsies reported a decrease in in vitro sliding velocity (Cuda et al. 1997), while later studies using human cardiac biopsy protein found an increase in sliding velocity (Palmiter et al. 2000). Studies using mouse α-cardiac myosin containing the R403Q mutation found an increase in velocity and also an increase in ATPase activity (Tyska et al. 2000). Lowey et al. (2008) subsequently showed that the effects of the R403Q mutation in mouse cardiac myosin depended on the isoform into which the mutation was introduced. In α-cardiac myosin, these authors saw an increase in both ATPase activity and velocity, whereas in the β-cardiac myosin, they observed no significant change in the velocity and actually a slight decrease in the ATPase activity (Lowey et al. 2008).

According to Eq. 1, increases in intrinsic force, velocity or ATPase activity will lead to the hyper-contractile phenotype at the molecular level, and this has been the general view of the molecular basis of hyper-contractility caused by HCM mutations. Recent work carried out on two early-onset HCM mutations using recombinant human β-cardiac myosin S1 indeed shows that all these parameters are significantly increased (25–90%) (Adhikari et al. 2016). However, the same experiments carried out on five typically adult-onset HCM mutations, including R403Q, using the same human β-cardiac myosin S1 construct, showed variable results with only modest changes compared to wild-type (WT) myosin (Kawana et al. 2017; Nag et al. 2015; Sommese et al. 2013). Moreover, some of these small changes contributed to hyper-contractility, others contributed to hypo-contractility, and some showed no change, making it difficult to assess the net effect of these mutations on power. It became clear that something was missing, and as described below, we believe that a primary effect of many HCM mutations in both myosin and MyBP-C is an increase in N_a_, resulting in more heads becoming functionally accessible for interaction with actin and offering a better explanation for the hyper-contractility observed clinically in the majority of adult onset HCM patients.

In order to map the known HCM mutations that are clearly pathogenic on the human β-cardiac myosin molecule we created homology models of the pre-stroke and post-stroke states of human β-cardiac myosin S1, with the full sequence of the human β-cardiac S1 heavy chain and the human ventricular ELC and RLC (Spudich 2015 ; Homburger et al. 2016). The templates used for the homology modeling were the crystal structure of the human β-cardiac myosin motor domain obtained by Winkelman et al. (2015) and the crystal structures of the lever arm containing the squid ELC and RLC. These homology models (HBCprestrokeS1.pdb and HBCpoststrokeS1.pdb) are available for download from our website (http://spudlab.stanford.edu/homology-models/).

**Fig. 4:**
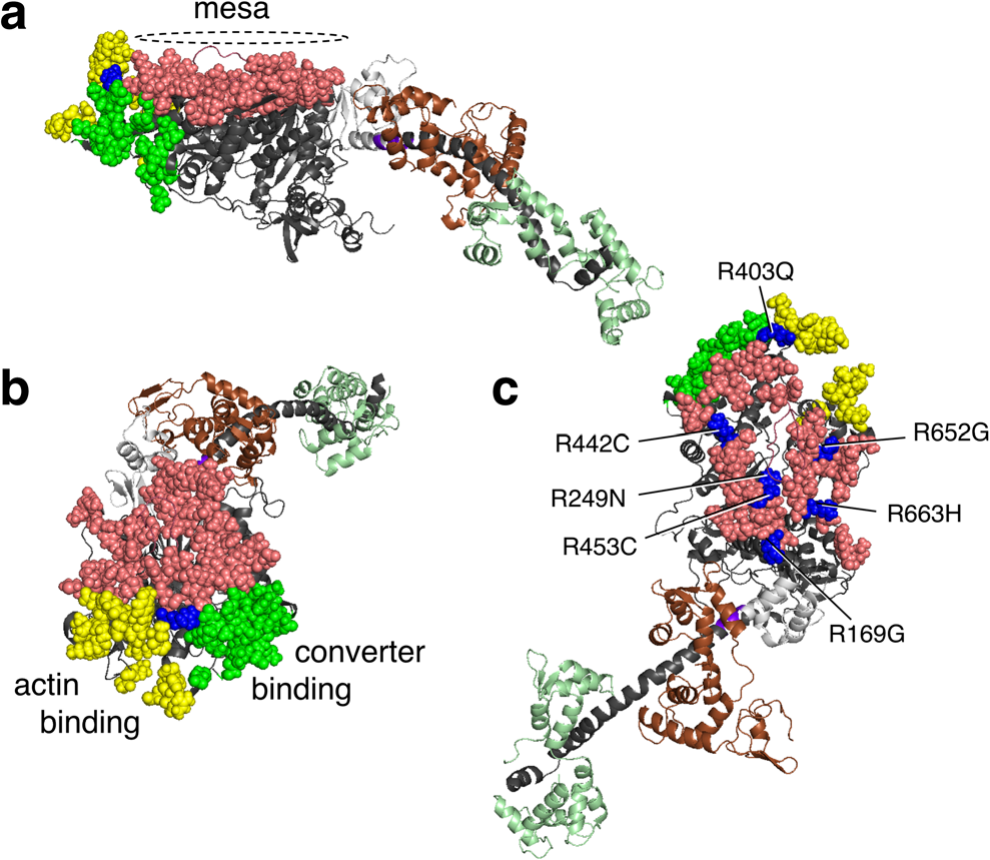
Human β-cardiac myosin structural model in its pre-stroke state showing the myosin mesa residues. **a** Side view of the myosin mesa with the mesa residues colored *pink*. The mesa surface is adjacent to two other surfaces of the S1 head, the actin binding domain (*yellow residues*) and a domain (*green residues*) that binds to the converter domain of another S1 head in a folded state of myosin discussed later in this review. The converter (*light gray*) is shown between the mesa and the light chain binding region. Between the converter and the light chain binding region is the pliant region (purple) which consists of six residues and is also a hot spot for HCM mutations. **b** These three surfaces form a pyramid-like structure with Arg-403 (*blue*) at its apex. **c** The positions of seven arginine residues on the mesa, all of which cause hypertrophic cardiomyopathy (HCM) when mutated

Examination of these human β-cardiac S1 models showed that a relatively flat surface on the S1 domain, which we termed the myosin mesa (Fig. 4a, pink residues), is highly conserved in cardiac myosin across species, including human β-cardiac myosin, and is a hot-spot for HCM mutations (Homburger et al. 2016; Spudich 2015). The mesa surface is at a steep angle to the actin-binding domain surface (Fig. 4a, yellow residues, as defined by Behrmann et al. 2012; Rayment et al. 1993; Schroder et al. 1993; von der Ecken et al. 2016) as well as to another surface on the S1 head (green residues) that plays into the story presented later in this review. Interestingly, the blue residue that is at the apex of the intersection of these three surfaces is Arg403, the first HCM mutation described more than 25 years ago (Geisterfer-Lowrance et al. 1990). In the first paper on the myosin mesa (Spudich 2015), only ‘HCM mutations’ that are clearly causative of hypertrophic cardiomyopathy were included, as defined by large numbers of independent diseased individuals carrying that mutation and/or families showing co-segregation of phenotypic disease and the ‘HCM mutation’. In particular, the myosin mesa has a positively charged cluster of arginine residues, all of which when mutated are clearly causative of HCM (Fig. 4c, blue residues). This cluster was suggested to act as a binding interface for another protein (Spudich 2015). Likely suspects because of their proximity in the sarcomere were MyBP-C and the proximal S2 region of the myosin (Spudich 2015; Spudich et al. 2016). The authors suggested that such binding interactions could sequester myosin heads in an ‘off state,’ thereby regulating the number of myosin heads functionally accessible (N_a_) for interaction with actin, and that a primary effect of HCM mutations could be to weaken such associations and thereby cause an increase in N_a_, thereby explaining the hyper-contractility observed clinically (Spudich 2015). This ‘mesa hypothesis’ was put on a firmer basis by the results of an analysis of the locations of 130 HCM mutations on human β-cardiac myosin that have been clearly shown to be causative of HCM clinically (Homburger et al. 2016; Nag et al. 2017). The findings of this extensive analysis revealed three hotspots in the myosin molecule for these HCM mutations, namely the mesa domain, the proximal S2 and the converter domain (Homburger et al. 2016). The residues that make up these domains, together with examples of HCM mutations in each domain, are shown in Table 1. About 70% of the myosin variants in the human population that map to the mesa region and 100% of those that map to the converter are disease producing (Homburger et al. 2016). Only approximately 20% of variants in other regions of the myosin motor domain are categorized as HCM pathogenic mutations. It should be noted that HCM mutations in residues other than those listed as mesa residues in Table 1 could well affect the proposed mesa-S2 and mesa-MyBP-C binding interactions.

**Table 1:**
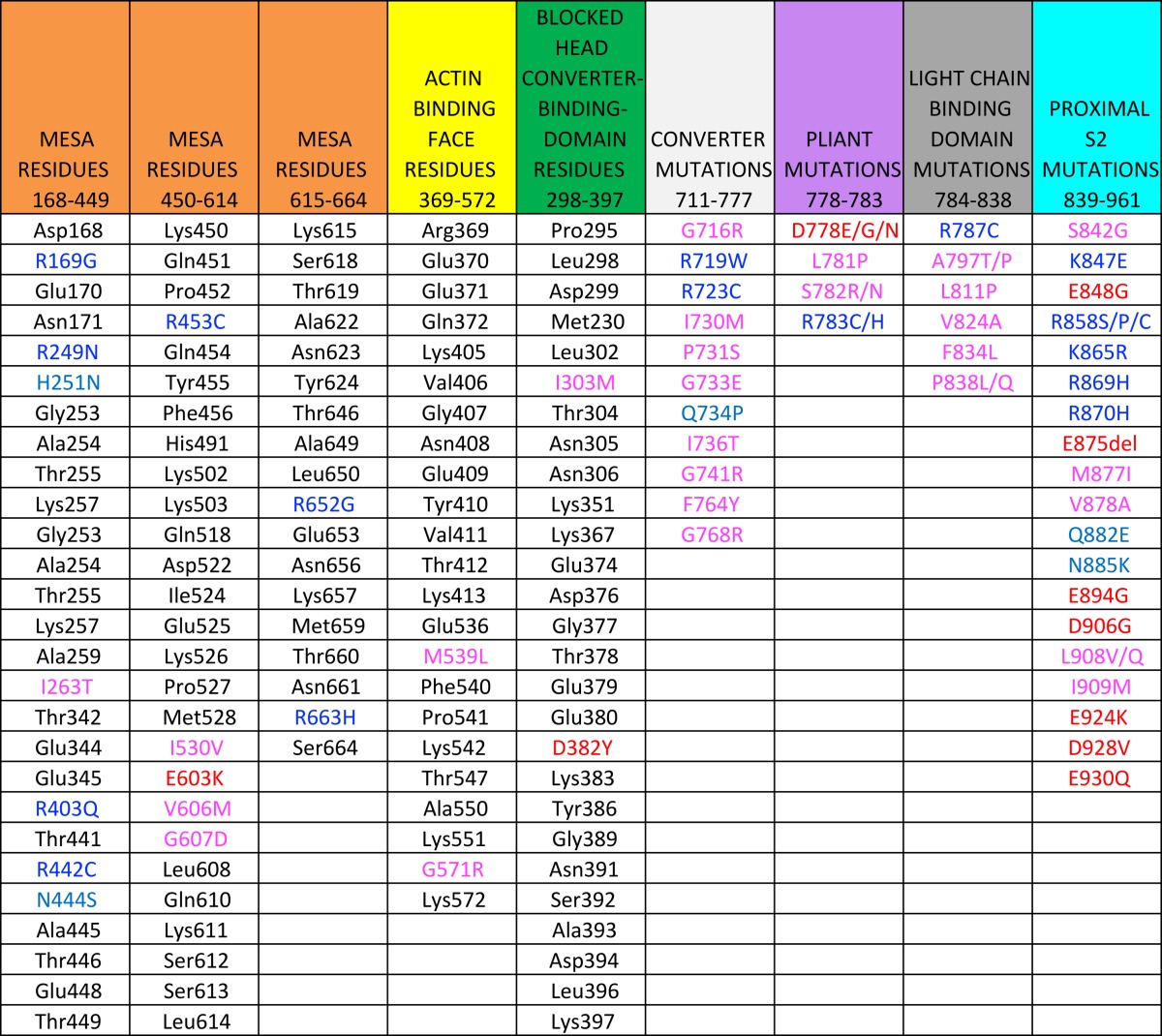
A list of residues forming the mesa, the actin-binding interface, and the blocked head interface that binds the converter of the free head are shown. The colored residues are HCM mutations

Residues identifed as HCM mutations are according to Homburger et al. 2016

Color coding of HCM residues: Blue, Arg and Lys; light blue, Gln, Asn, His; red, Glu and Asp; magenta, all non-charged residues. Note that the mesa is rich in positively-charged HCM residues while the proximal S2 is rich in negatively-charged HCM residues. Note that only residues mutated in HCM are shown in the last 4 columns

## A folded-back state of myosin known as the interacting-head motif is tailor-made for regulation of N_a_

**Fig. 5:**
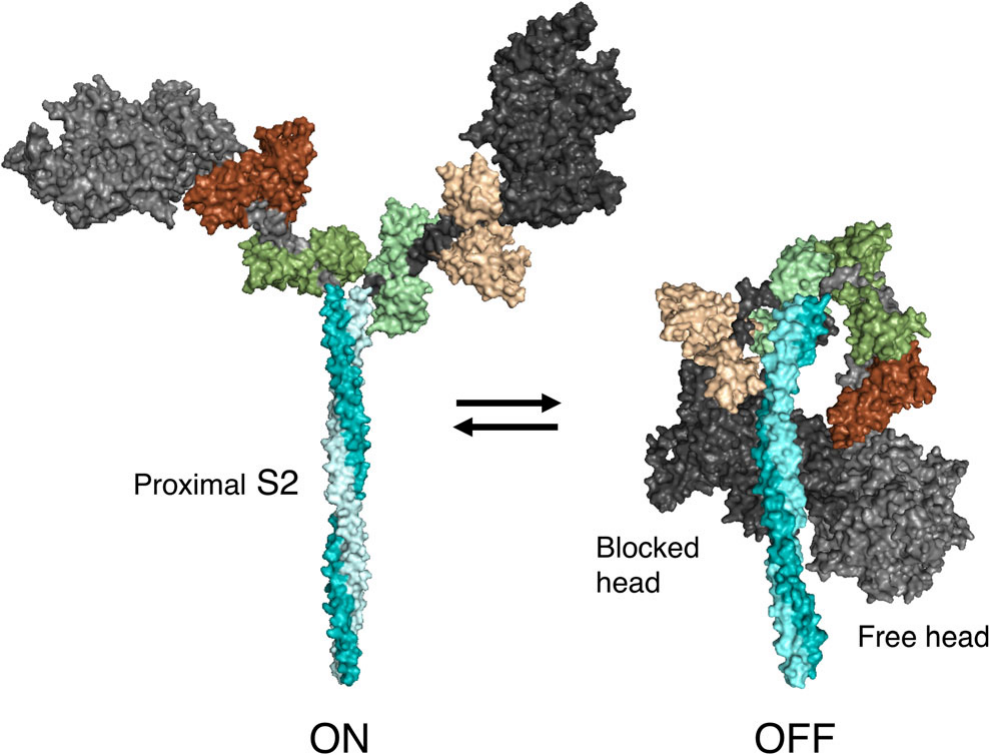
Structural models of the open ‘on state’ and the ‘off state’ of the interacting-heads motif (IHM) of human β-cardiac myosin. The templates used to model the open state were obtained from the human β-cardiac myosin motor domain solved by Winkelmann et al. (2015), supplemented with the rigor structure from the squid myosin motor domain (Yang et al. 2007), as described in Nag et al. (2017). The template used to model the closed state is based on the three-dimensional (3D) reconstruction of tarantula skeletal myosin thick filaments by Alamo et al. (2016) (PDB 3JBH). A short version of myosin HMM, showing only 126 residues of the coiled-coil S2 domain, is illustrated in its ‘on and off states’, which are in equilibrium. The back view (named from the 3D reconstruction of the tarantula thick filament; this side faces the myosin bipolar thick filament) of the IHM state is shown

Intramolecular interactions favoring a folded state of myosin (Fig. 5) have been observed in isolated HMM in solution (Burgess et al. 2007; Jung et al. 2008b, 2011; Wendt et al. 1999, 2001), myosin molecules in solution (Burgess et al. 2007; Jung et al. 2008a, b, 2011) and intact thick filaments (Al-Khayat et al. 2013; Woodhead et al. 2005; Zhao et al. 2009; Zoghbi et al. 2008). The latter also reveals intermolecular interactions among neighboring myosin heads (Al-Khayat et al. 2013; Alamo et al. 2016; Gonzalez-Sola et al. 2014; Pinto et al. 2012; Woodhead et al. 2005; Zhao et al. 2009; Zoghbi et al. 2008). The first reports of a state of myosin in which the two heads interact asymmetrically were from 2D-crystalline arrays of unphosphorylated smooth muscle myosin (Wendt et al. 1999; 2001). Using cryo electron microscopy (EM), Wendt et al. (2001) described a three-dimensional (3D), 2.0-nm resolution structure of this folded state of myosin, later named the interacting heads motif (IHM) (Alamo et al. 2008). In this folded ‘off state’ of the motor domains, a binding surface of the ‘blocked head’ (so called because the actin-binding domain is sequestered in the folded molecule) binds to the converter domain of the ‘free head’ (so called because the actin-binding domain is not sequestered in the folded molecule). Observation of the folded-back IHM structure for other myosins indicates that the blocked head may also be in structural contact with proximal S2 (Adhikari et al. 2016; Alamo et al. 2008, 2016; Burgess et al. 2007; Jung et al. 2008a; Nag et al. 2017; Woodhead et al. 2005) (Fig. 5). This IHM of myosin has been observed in several classes of myosins, including anemone myosin (Sulbaran et al. 2015) and striated muscle myosins (Jung et al. 2008b). In full-length smooth muscle myosin, the LMM region can further fold back onto the head region (Burgess et al. 2007; Jung et al. 2008a, b).

The IHM structure has been notable in images of intact thick filaments isolated from a variety of types of muscle. Thus, low-resolution thick filament structures have been observed in EM studies of tarantula skeletal muscle (Woodhead et al. 2005), *Limulus* (Zhao et al. 2009), scallop striated muscle (Woodhead et al. 2013), scorpion skeletal muscle (Pinto et al. 2012), zebrafish cardiac muscle (Gonzalez-Sola et al. 2014), *Schistosoma mansoni* smooth muscle (Sulbaran et al. 2015) and vertebrate cardiac muscle (Al-Khayat et al. 2013; Zoghbi et al. 2008). A perpendicular-type of IHM has been reported for *Lethocerus* indirect flight muscle (Hu et al. 2016). The highest resolution achieved to date for the region of myosin heads is 2.0 nm for the conventional parallel-type of IHM in tarantula (Alamo et al. 2008) and 2.0 nm for the conventional perpendicular-type IHM in *Lethocerus* (Hu et al. 2016).

Three-dimensional reconstruction of the electron density maps from isolated thick filaments confirmed the presence of a helical array of myosin heads, also known as crowns, along the circumference of the thick filament. The crowns are separated by an approximately 14.5-nm axial repeat with a twist of 30° in the tarantula thick filament (Woodhead et al. 2005). Three successive crowns form the basic repeating unit in the helical array of heads in the thick filament. In cardiac thick filaments, three crowns within a 42.9-nm repeat are arranged in a perturbed helical fashion. Inter-crown distances have been observed to be different than 14.3 nm in between certain crowns (Al-Khayat et al. 2013). Furthermore, there is a deviation in azimuthal position from an ideal helical array. Additionally, extra densities, possibly corresponding to the positions of titin and MyBP-C, were described in a mouse cardiac muscle thick filament reconstruction (Zoghbi et al. 2008) and later in a human cardiac thick filament reconstruction (Al-Khayat et al. 2013). According to the model of Al-Khayat et al. (2013), the human cardiac thick filament has 11 domains of titin in every 42.9-nm repeat where two pairs of such titin molecules are present adjacent to three crowns, and the three C-terminal domains of MyBP-C lie close to one of the three crowns. Two of the three crowns of the 42.9-nm repeat in the cardiac thick filament have the myosin heads interacting asymmetrically as in the folded state, whereas the third one has a relatively mobile conformation (Al-Khayat et al. 2013; Zoghbi et al. 2008). This mobile conformation may relate to the swaying head concept proposed by Brito et al. (2011) on the basis of structural considerations which suggested that the free heads are less strongly bound to the filament backbone and may oscillate occasionally between the attached and detached states (swaying heads).

Atomic fitting of myosin heads into the 3D reconstruction of relaxed tarantula thick filaments suggests that intramolecular head–head interactions are present within the crowns as well as intermolecular interactions (Woodhead et al. 2005; Alamo et al. 2008, 2016). Intermolecular interactions between heads of adjacent crowns has been observed in a variety of thick filament types (Alamo et al. 2008; Al-Khayat et al. 2013; Gonzalez-Sola et al. 2014; Pinto et al. 2012; Sulbarán et al. 2015; Woodhead et al. 2005; Zhao et al. 2009; Zoghbi et al. 2008).

To summarize to this point, Alamo et al. (2008) has reported the highest resolution (2.0 nm) 3D reconstruction achieved to date for the IHM structure of frozen-hydrated thick filaments (tarantula), and later studies from that group (Alamo et al. 2016) based on that 3D reconstruction have been very useful for understanding not only tarantula thick filament structure and function, but they have also had important implications for other invertebrate and vertebrate skeletal muscles. While higher average resolutions of 1.3 and 0.6 nm have been achieved for the tarantula (Yang et al. 2016) and *Lethocerus* thick filaments (Hu et al. 2016), respectively, their actual resolutions in the region of the myosin IHMs unfortunately are still 2.0 nm. The prospect of increasing this limiting resolution of 2 nm on the head regions of the thick filament is not promising since the disorder stemming from the free swaying heads is intrinsically reflected after the rapid freezing step (Yang et al. 2016). Although it is possible that the swaying heads could be locked down by treatment with, for example, blebbistatin, which stabilizes the IHM (Zhao et al. 2009), as already mentioned, not all IHM structures in the thick filament are identical, which severely limits the resolution that can be obtained. A higher resolution structure of the IHM per se is more likely to come from purified myosins containing two heads and the proximal S2, again stabilized in some way in the IHM state. Thus, while admittedly difficult, one future direction that is urgently needed in this field is to obtain a true atomic-resolution structure of the IHM, either by high-resolution EM or by X-ray crystallography. From the point of view of the effects of HCM mutations on the IHM structure, the high-resolution structure of the human β-cardiac myosin IHM is needed.

A step in this direction is the work of Blankenfeldt et al. (2006). These investigators solved the crystal structure of the proximal S2 region of human β-cardiac myosin, pointing out three distinct clusters or rings, Rings 1, 2 and 3, of negative charge along the proximal S2 interrupted by zones of neutral or moderately positive character. They fitted the proximal S2 crystal structure to the tarantula IHM structure of Woodhead et al. (2005) and drew attention to potential interactions between the blocked head and the S2 Ring 1 that possibly involve the positively charged loop 2 of the blocked head and the free head and the S2 Ring 2. These authors further showed that the disordered nature of the head–rod junction is important for the formation of the IHM folded structure.

## The IHM state is regulated by RLC phosphorylation

It has long been known that phosphorylation–dephosphorylation of the RLC of smooth muscle myosin is an on–off switch for smooth muscle myosin activity (Lowey and Trybus 2010). This regulation has been observed at the molecular level by monitoring the catalytic efficiency of myosin as a function of phosphorylation of the RLC. Smooth muscle RLC phosphorylation has been shown to release the myosin heads from the folded-back IHM state into an ‘on state’ available for actin interaction (Wendt et al. 1999). The dephosphorylated smooth muscle myosin has very low actin-activated ATPase activity and cannot translocate actin in an in vitro motility assay (Trybus et al. 1997). Phosphorylation of even one of the heads of smooth muscle HMM can lead to a functional motor (Rovner et al. 2006; Walcott et al. 2009). Single-headed smooth muscle HMM does not retain the regulation of the two-headed construct, indicating that the folded state IHM requires the interaction of both of the heads of myosin (Cremo et al. 1995, 2001).

Although striated muscle myosins are not regulated in an on–off fashion by RLC phosphorylation, it is clear that RLC phosphorylation does affect their structure and function. For example, the myosin heads of skinned rabbit skeletal muscle fibers become more mobile when the RLC is phosphorylated at its serine 15 residue by the calmodulin-myosin light chain kinase (MLCK) complex in the presence of Ca^2+^ (Levine et al. 1996). RLC phosphorylation also has an impact on the activity of cardiac muscle myosin. RLC phosphorylation is important for normal heart function, and dephosphorylation of RLC can lead to hypertrophy of the heart (Huang et al. 2008). In the human cardiac sarcomere, approximately 40% of the RLCs are estimated to be phosphorylated at Ser-15 by balanced activities of MLCK and myosin light chain phosphatase (Toepfer et al. 2013). In rat cardiac trabeculae, RLC phosphorylation increases the velocity of shortening of the muscle, isometric force production and power generation (Toepfer et al. 2013). Polarized fluorescence intensity measurements in ventricular trabeculae of the rat heart have also revealed that phosphorylation of the RLC shifts the equilibrium between folded and open states toward more open heads of myosin (Kampourakis and Irving 2015).

Important findings on the role of phosphorylation and the control of the number of myosin heads that are functionally available for interaction with actin (N_a_; Eq. 1) come from studies of tarantula skeletal myosin (Alamo et al. 2008, 2015; Brito et al. 2011; Craig et al. 1987; Espinoza-Fonseca et al. 2015; Padron et al. 1991; Sulbaran et al. 2013). As early as 1987, Craig, Padron and Kendrick-Jones (Craig et al. 1987) showed that phosphorylation of tarantula skeletal myosin is accompanied by potentiation of the actin activation of the myosin ATPase activity and by the loss of order of the helical crossbridge arrangement characteristic of the IHM state in relaxed tarantula thick filaments. These authors suggested that in the relaxed state, when the RLC are not phosphorylated, the myosin heads are held down on the filament backbone in what is now considered to be the IHM state and that phosphorylation of the RLC cause the crossbridges to become more loosely associated, with the filament backbone giving rise to the observed changes and facilitating crossbridge interaction with actin. Then in 1991, Padron and colleagues (Padron et al. 1991) showed changes in the equatorial X-ray diffraction patterns of tarantula muscles in the phosphorylated state that supported the movement of myosin heads away from the backbone of the filament. The results of further study by these same authors led to their suggesting that the structural differences between the blocked and free heads in the IHM could preset the order in which the two heads are released upon phosphorylation (Alamo et al. 2008). The free head would be the one conformationally and properly located to be released first to interact with actin, followed by the blocked head (Alamo et al. 2008; Brito et al. 2011). Thus, the free heads were suggested to be less strongly bound to the filament backbone and may oscillate occasionally between the attached and detached states (swaying heads). It was postulated that once MLCK becomes activated, it phosphorylates free heads, which then renders the free heads fully mobile, exposing the blocked head phosphorylation site to MLCK. This would release the blocked heads, allowing their interaction with actin. According to this model, twitch force would be produced by the rapid interaction of swaying free heads with activated thin filaments, although it should be noted that for the free heads to be able to interact with actin, the S2 would have to be released from the shaft of the thick filament to allow the heads to reach the actin. Further experimental evidence supporting the presence of swaying heads and a cooperative phosphorylation mechanism for activation of tarantula thick filaments were presented in subsequent papers on the tarantula IHM state (Alamo et al. 2015, 2016; Espinoza-Fonseca et al. 2015; Sulbaran et al. 2013; for review, see Vandenboom 2016). With respect to the focus of the present review, which is the cardiac system, it will be very important to examine the details of RLC phosphorylation control of the cardiac sarcomere to see if it is the same as that in the tarantula skeletal muscle.

A beginning of such studies on the cardiac system is the functional effect of RLC phosphorylation using two-headed human β-cardiac myosin with two different lengths of the coiled-coil S2 tail region (Nag et al. 2017). The two HMM constructs, i.e. 2-hep HMM and 25-hep HMM, have two heptad repeats and 25 heptad repeats of the S2 tail region, respectively. 25-hep HMM shows an inhibition of its ATPase of approximately 40% when its RLCs are de-phosphorylated. In contrast, 2-hep HMM shows no effect of the de-phosphorylation of Ser-15 of human cardiac RLC on the maximal ATPase rate in actin-activated ATPase assays, indicating that head–tail interactions lower activity in the 25-hep construct. Importantly, the phosphorylation of RLCs in 25-hep HMM leads to a maximal activity (k_cat_ of approx. 2.5 s^−1^) that is the same as that of the 2-hep HMM (k_cat_ of approx. 2.5 s^−1^) and of a truncated form of human β-cardiac myosin S1 lacking the RLC (short S1, or sS1; k_cat_ of approx. 2.5 s^−1^) (Adhikari et al. 2016; Kawana et al. 2017), where head–tail interaction is not possible. This result fits well with the results of studies of Trybus et al. (1997) who showed that the minimal size at which smooth muscle myosin molecules are capable of regulation via light chain phosphorylation includes the presence of a length of S2 approximately equal to the size of the myosin head. These authors concluded that the myosin S2 mediates specific interactions with the head that are required to obtain the completely inactive state of smooth muscle myosin.

In addition to demonstrating control of contractility by RLC phosphorylation, it has been shown that mechanical force applied on skeletal myosin thick filaments can regulate the number of active heads (Linari et al. 2015). As the load on thick filaments increases, more heads are liberated from the folded IHM state, resulting in an increase in the number of active heads. A similar mechanism was also shown to operate in cardiac muscle wherein during transition from diastole to systole, the number of myosin motors recruited from the IHM state are related to the systolic force, which is in turn dependent on the sarcomere length (Reconditi et al. 2017; Zhang et al. 2017). Reconditi et al. (2017) reveal that in an intact cell at rest, most myosin motors are in an IHM ‘off state’ and that the number of myosin motors switching on increases with the stress on the thick filament. This study also hints at a possible link between HCM-causing mutations which affect the intra-and intermolecular interactions that hold the IHM state of myosin motors and the hyper-contractility observed in HCM patients. A loss in the stress-sensing mechanism of the thick filament by mutations in myosin or myosin binding protein C can adversely affect the stability of the IHM state of myosin motors. On the other hand, it has also been shown that sarcomere length-dependent structural changes in the thick and thin filaments are related to the passive force generated by titin (Ait-Mou et al. 2016; Zhang et al. 2017). Based on this concept, it can be envisioned why some titin mutations tend to cause DCM. A number of missense mutations would decrease the stiffness of titin, which could result in the loss of titin’s ability to activate the thick filament and in turn lead to a reduction in the number of functionally accessible myosin heads and ultimately to DCM.

## Myosin binding protein C may be involved in the IHM state

### MyBP-C is a linear polymer localized along the myosin thick filament in muscle

The location of MyBP-C in the thick filament was first revealed by immuno-EM, visible as eight or nine transverse stripes (C-zone) in each half of the A-band (Craig and Offer 1976; Lee et al. 2015; Luther et al. 2008) (Fig. 1). These stripes are approximately 43 nm apart, the same as the true repeat distance between myosins along the thick filament. MyBP-C is a structural component of the thick filament and expressed as three isoforms in human muscle: the fast skeletal isoform, the slow skeletal isoform and the cardiac isoform. The cardiac MyBP-C is a 140-kDa protein, with a length and diameter of approximately 40 and 2–3 nm, respectively, consisting of 11 subdomains, eight of which belong to the immunoglobulin family (C0–C5, C8 and C10) and three to the fibronectin type-III family (C6, C7, C9), plus a proline–alanine-rich domain (PA) between the C0 and C1 domains and a phosphorylatable M domain between the C1 and C2 domains (Fig. 3). MyBP-C is thought to help in the regular organization of the thick filaments.

The cardiac isoform when compared to the skeletal isoform has characteristic structural additions which may hint at a special role of the cardiac MyBP-C. These additions are the globular C0 domain at the N-terminus, the phosphorylatable M-domain and a 28 amino acid-charged loop added to the C5 domain (Flashman et al. 2004). The phosphorylatable motif, referred to as the M-domain, has four serines that can be phosphorylated upon adrenergic stimulation (Fig. 3). The M-domain is a target of protein kinase A (PKA) (Gautel et al. 1995; Mohamed et al. 1998), protein kinase C (Mohamed et al. 1998; Xiao et al. 2007), protein kinase D (Bardswell et al. 2010; Dirkx et al. 2012), p90 ribosomal S6 kinase (Cuello et al. 2011) and Ca^+2^/calmodulin-dependent kinase II (Gautel et al. 1995; Sadayappan et al. 2011). Additionally, the flexible proline/alanine-rich linker called the PA loop also exists in cardiac MyBP-C between the C0 and C1 domains. For the remainder of this review, the term MyBP-C is used to refer to the cardiac isoform.

There have been reports that sequence differences of the PA loop between various species can modulate the actin–myosin interaction and that the percentage of prolines and alanines in the PA loop are inversely proportional to the heart rate (Shaffer et al. 2010). The PA loop is also rich in negatively charged residues, which may hint at a role of the loop in acting as a binding interface. Indeed, a recent study by Colson et al. (2016) demonstrated that phosphorylation-dependent allosteric changes do propagate between the M-domain and the PA loop.

MyBP-C has a number of post-translational modifications apart from phosphorylation, including acetylation (Ge et al. 2009; Govindan et al. 2012), *S*-glutathionylation (Patel et al. 2013), citrullination (Fert-Bober and Sokolove 2014), carbonylation (Aryal et al. 2014) and *S*-nitrosylation (Kohr et al. 2011). The roles of these post-translational modifications are not known and is an active area of research. Recent evidence suggests that Ca^2+^ induces structural changes in MyBP-C that override the impact of phosphorylation (Previs et al. 2015, 2016). These studies, performed with isolated N-terminal fragments of MyBP-C, show that the phosphorylation effects of MyBP-C are maximum when the Ca^2+^ levels are low. There is also evidence that the M-domain of MyBP-C can bind calmodulin in a Ca^2+^-dependent manner and that it may represent a structural connector between Ca^2+^, phosphorylation and other signaling pathways (Lu et al. 2012; Michie et al. 2016).

### MyBP-C has been shown to interact with both actin and myosin and is implicated in several regulatory functions

Myosin binding protein C is tightly bound to the myosin thick filament backbone or the LMM region by way of its C-terminus (C8–C10) (Flashman et al. 2007; Miyamoto et al. 1999). The C-terminus also interacts with titin in the thick filament (Freiburg and Gautel 1996). The C0–C7 region of MyBP-C is thought to extend out from the thick filament, and there is evidence supporting its modulation of contraction by interaction with both myosin and actin filaments (Kampourakis et al. 2014). The N-terminal domain of MyBP-C (C0–C2) binds to the regulated actin filament, where it is thought to activate contraction by facilitating the movement of tropomyosin away from its blocked state for myosin binding (Mun et al. 2014). A considerable amount of work has been devoted to the interaction of the N-terminal domain of MyBP-C with the thin filament (Kulikovskaya et al. 2003; Rybakova et al. 2011; Shaffer et al. 2009; Squire et al. 2003; Weith et al. 2012), and this aspect of muscle biology has been the focus of recent reviews (Craig et al. 2014; van Dijk et al. 2014).

**Fig. 6:**
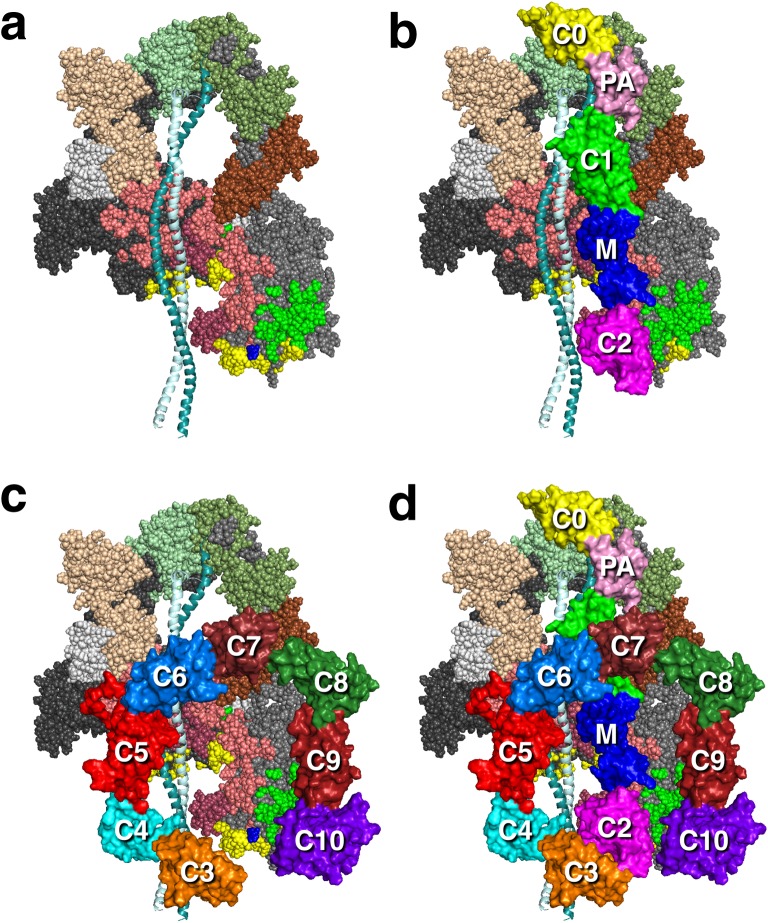
Working structural models of the IHM state of human β-cardiac myosin with MyBP-C fragments bound. **a** The heavy chain residues of the blocked S1 head (*on the left*) are *colored pink* (mesa residues), *reddish-brown* (loop 2), *yellow* (actin-binding domain), bright green (converter binding domain; barely visible), *light gray* (converter) and *dark gray* (all remaining residues). The ELC is colored *light brown* and the RLC is light green. The color scheme of the free head (*on the right*) is the same as that of the blocked head, except the main body of the heavy chain is colored medium grey. The green residues correspond to those residues on the blocked head that interact with the converter domain of the free head. The ELC is colored *dark brown* and the RLC is *dark green*. **b** A possible orientation of the C0–C2 domains of MyBP-C is shown, with potential interactions between the C0–C2 domains and the mesa of the free head (*on the right*) and illustrating potential interactions between proximal S2 and C1–C2 (Gruen and Gautel 1999). The *yellow* C0 domain is bound to the RLCs (Ratti et al. 2011) and the proline–alanine-rich domain (*PA*; *light pink*) connects to the C1 (*green*)–M (*blue*)–C2 (*magenta*) domains, which are on this backside view of the IHM complex. **c** A possible orientation of the C3–C10 domains of MyBP-C is shown, with potential interactions between the C5–C6 domains and the mesa of the blocked head (*on the left*) and illustrating potential interactions between proximal S2 and the C3–C6 domains. **d** Hypothetical model of the interaction of full-length MyBP-C with the IHM state. These structures are working models for experiments going forward

The interaction of MyBP-C with myosin (Starr and Offer 1978), in contrast, has received relatively less attention. Studies by Gautel and Pfuhl and their colleagues have demonstrated that the cardiac-specific C0 domain binds to the myosin RLC with a stoichiometry of 1 C0 domain to 2 RLCs (Ratti et al. 2011). These authors have also shown that the C1 domain binds to the proximal part of S2, very close to the light chains (Ababou et al. 2008). Finally, the C1–C2 construct binds to proximal S2 with a K_d_ of 5 μM (Gruen and Gautel 1999). These results are schematically depicted in the theoretical working model of the IHM state with MyBP-C C0–C2 bound shown in Fig. 6b.

It has been suggested that the N-terminal region of MyBP-C might switch positions between actin and myosin binding as a regulatory mechanism (Kampourakis et al. 2014; Moss et al. 2015). The interactions of C0–C2 with myosin are thought to inhibit the function of the myosin heads, and HCM mutations have been suggested to affect this regulation (Ababou et al. 2007, 2008; Gruen and Gautel 1999; Gruen et al. 1999; Ratti et al. 2011). By characterizing the effects of the C1–C2 MyBP-C fragment on the structures of the thin and thick filaments in contracting heart muscle cells, Kampourakis et al. (2014) showed that MyBP-C stabilizes the ‘on state’ of thin filaments and the ‘off state’ of thick filaments. Their results are consistent with a model for the control of heart muscle contraction in which the regulatory functions of the thin and thick filaments are coordinated by MyBP-C.

As shown recently, human de-phosphorylated cardiac MyBP-C binds directly to human β-cardiac short S1 (sS1), which lacks the RLC, with a K_d_ of approximately 15–20 μM, and phosphorylation of the MyBP-C with PKA reduces the affinity of the binding significantly (Nag et al. 2017). Similar but somewhat weaker binding (K_d_ = approx. 35 μM) was seen with the de-phosphorylated C0–C2 domain alone. This was the first experimental evidence of full-length MyBP-C or the N-terminal fragment of MyBP-C binding the sS1 region of myosin in a phosphorylation-dependent manner, and it suggests that MyBP-C can bind both the S1 and S2 regions of myosin and thus possibly hold the heads in the IHM state on the thick filament (Fig. 6). The equilibrium between sequestered and free heads could then be shifted toward free heads upon phosphorylation of the MyBP-C.

**Fig. 7:**
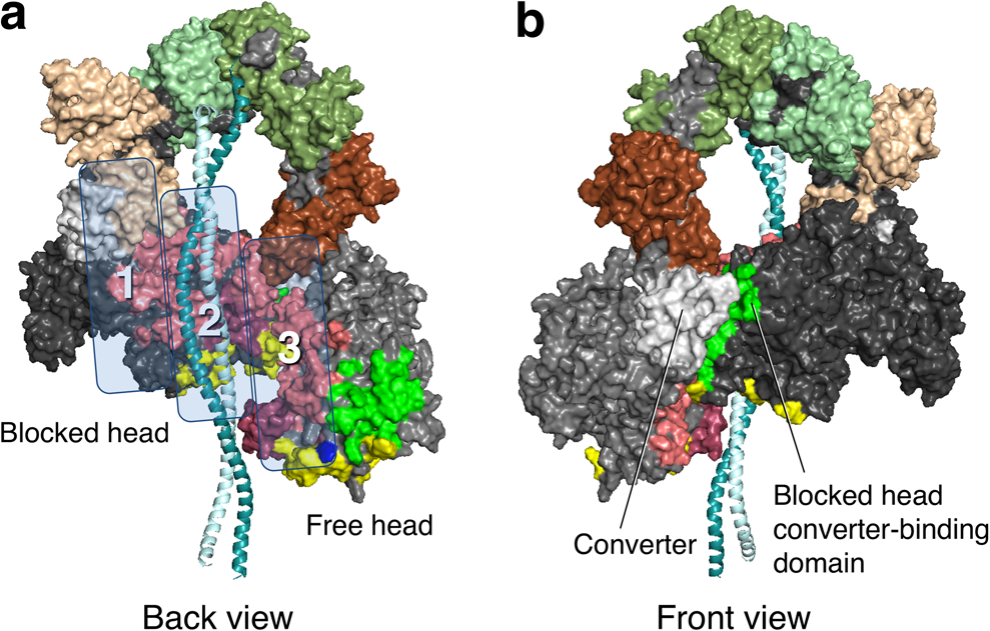
Structural model of the IHM state of human β-cardiac myosin viewed from the back and the front. **a** Coloring of domains is the same as in Fig. 6. The back view shows the proximal S2 associating with the mesa of the blocked head in zone 2. Zones 1 and 3 are possible interaction sites for domains of MyBP-C (see Fig. 6). **b** The front view shows the blocked head converter-binding domain (*green*) binding to the converter (*light grey*) of the free head (*on the left*)

EM has revealed the full-length MyBP-C to be V-shaped, with the position of the kink near to the C4 domain (Previs et al. 2016). In our homology-modeled human β-cardiac myosin IHM structure, we have modeled a folded-back full-length MyBP-C molecule such that the central C5–C6 domains bind at the interface of the blocked head S1 and proximal S2 (Fig. 6c). Such a structure would be consistent with the observation that full-length MyBP-C binds more tightly to the sS1 fragment than the C0–C2 fragment (Nag et al. 2017). In this highly hypothetical model, the C8–C10 domains would be available for binding to the LMM shaft of the thick filament. This working model roughly divides the mesa domains in the IHM state into three zones, with zone 1 consisting of the C5–C6-blocked head interaction, zone 2 consisting of the S1–S2 interaction, and zone 3 consisting of the C0–C2-free head interaction (Fig. 7). We have made three different models of MyBP-C bound to the IHM structure (see http://spudlab.stanford.edu/homology-models/, where they can be downloaded as pdb files). The third model, shown in Fig. 6, is most consistent with available structural data (Al-Khayat et al. 2013; Lee et al. 2015; Zoghbi et al. 2008). We emphasize that these models serve as working hypotheses to guide future experiments and should not be considered to be actual structures. More models need to be considered. For example, one binding study would suggest that C2 may be closer to the N-terminal part of the proximal S2 than our current models depict, but the K_d_ of that interaction was only 1.1 mM (Ababou et al. 2007). Our models focus on possible conformations of MyBP-C bound to myosin holding heads in a sequestered state. The other mode of regulation by MyBP-C is thought to be via its interaction with the thin filament. A recent structural study by Luther et al. using electron tomography of frog sartorius skeletal muscle shows convincingly that, under the relaxing conditions (without Ca^2+^) used, MyBP-C reaches across the gap between the thick and thin filaments (Luther et al. 2011). The phosphorylation status of the skeletal MyBP-C or of the myosin RLCs was not described, and further structural studies using this approach under a variety of phosphorylation states may provide interesting results. Also, it is becoming increasingly clear that Ca^2+^ plays a role in fine-tuning the conformation of MyBP-C (Previs et al. 2015, 2016).

Transgenic mouse studies have yielded invaluable insights into the workings of MyBP-C. Targeted knockout (MyBP-C ^−/−^) mice do not show embryonic lethality, but develop severe cardiac hypertrophy at around 3 weeks after birth. These hearts have myocyte disarray and increased fibrosis, typical signs of HCM (Carrier et al. 2004; Cazorla et al. 2006; Harris et al. 2002). Fiber studies from these mice demonstrated that they had increased crossbridge cycling rates and increased power output (Korte et al. 2003; Stelzer et al. 2006a, b). Structural data based on low-angle X-ray scattering and EM studies also revealed that the homozygous knockout mice have myosin heads that are more disordered than those of the WT mouse and that the myosin heads move out azimuthally away from the thick filament surface upon loss of MyBP-C (Colson et al. 2007). A very recent EM study (Kensler et al. 2017) imaged isolated thick filaments from transgenic mouse hearts that had three of the phosphorylatable serine residues of the M-domain replaced either with alanine or aspartic acid to mimic the non-phosphorylated or the fully phosphorylated state of MyBP-C, respectively. This work demonstrated that the thick filaments from the phospho-deficient hearts had highly ordered presumed ‘off-state’ myosin heads, while the thick filaments from phosphomimetic hearts had more disordered myosin heads, consistent with their switch to an ‘on state’ for interaction with actin. All these data support the notion that MyBP-C modulates the actin–myosin interaction partly through interaction of MyBP-C with myosin by tethering myosin heads to the thick filament, and that this tethering is weakened upon the phosphorylation of MyBP-C.

Further studies are required in all areas of MyBP-C regulation of contraction. Particular attention needs to be given to the roles of the central C3–C7 domains, especially in the context of the novel 28 amino acid insertion in the C5 domain that is specific to cardiac MyBP-C. Of critical importance is a high-resolution cryo-EM-based or X-ray crystallographic structure of the entire MyBP-C bound to myosin. Such a structure will yield essential insights into the nature of molecular interactions in the proposed IHM complex.

## A super-relaxed state of myosin has been postulated to be related to the IHM state

Cooke and colleagues discovered the existence of a novel state of myosin molecules in striated muscle fibers which they termed the super-relaxed state (SRX). This state was initially discovered in rabbit skeletal muscle and thereafter also seen in rabbit cardiac, tarantula skeletal and mouse cardiac fibers (Hooijman et al. 2011; McNamara et al. 2016; Naber et al. 2011; Stewart et al. 2010). The hallmark of this state is that myosin molecules in the SRX state have an extremely slow release of nucleotide from the active site. An appealing hypothesis is that the SRX state is related to the IHM state seen in EM studies (Alamo et al. 2016; Cooke 2011; Hooijman et al. 2011; Nogara et al. 2016b; Wilson et al. 2014), as discussed in more detail below.

Thus, myosin is thought to exist in three states in the sarcomere. The first is the active cycling state of myosin which interacts with the thin filament and has a rapid ATP turnover time of <1 s. The second state, often called the disordered relaxed myosin, has an intermediate ATP turnover time of <30 s. In this state the myosin is detached from actin and cycles ATP at a basal rate. The third, or SRX state, is the state which has very slow ATP turnover times of more than 100 s. In cardiac sarcomeres the approximate ATP turnover time in the SRX state is approximately 140 s per myosin (Hooijman et al. 2011).

There is a key difference between the SRX of skeletal and cardiac sarcomeres. In the skeletal fiber, upon activation by Ca^2+^, the SRX state is completely abolished, while in the cardiac fiber even after activation the slow phase of ATP turnover is still observed (Hooijman et al. 2011; Stewart et al. 2010). This hints at a possible fundamental difference in the allosteric communication that occurs between different myosin molecules within a skeletal versus a cardiac thick filament. In the skeletal sarcomere, it has been shown that a few tightly actin-bound heads of myosin can generate thick filament stress which in turn activates heads out from the SRX into the disordered state which are then recruited for contraction (Linari et al. 2015). This cooperative mechanism may explain the disappearance of SRX upon activation of skeletal fibers, but such a mechanism may not operate in cardiac muscle. The skeletal muscle needs to generate instantaneous force upon activation, while cardiac muscles need to undergo repetitive cycles of contraction and relaxation during every heartbeat. Thus, in the case of the heart, the SRX may provide a cardioprotective role by sequestering myosin heads and thus minimizing the energy usage of the heart, all the while keeping an important reserve of heads available when needed (Hooijman et al. 2011; McNamara et al. 2015).

It is important to note that there have been no studies in vertebrate striated fibers that directly demonstrate any structural corollary of the SRX state. Cooke and his colleagues (Alamo et al. 2016; Nogara et al. 2016b) have made the reasonable and appealing proposal that the SRX is related to the folded-back sequestered IHM state seen by others in EM studies. This relationship seems highly likely, but SRX has only been described biochemically in skinned striated muscle fibers, where other proteins associated with the thick filament could easily be playing a role in reducing basal level ATPase rates to the SRX level. Alamo et al. (2016) have hypothesized that conserved intramolecular interactions that maintain the myosin IHM state can explain the structural basis of the tarantula muscle super-relaxed state. This hypothesis needs to be tested biochemically, however, since detailed molecular interactions in the IHM remain hypothetical with a 2-nm resolution map and will have to await a high-resolution (3 Å or better) structure of the IHM. Data are available on purified de-phosphorylated smooth muscle HMM, showing a very slow ATPase (<0.004 s^−1^) (Cremo et al. 1995), but phosphorylation/dephosphorylation is an on/off switch for smooth muscle, unlike striated muscle. A recent study by Rohde et al. (2017) demonstrated a marginal increase in the basal ATPase of bovine cardiac HMM at 25 mM KCl versus 0 mM KCl which may hint at disruption of the IHM by increased ionic strength. However, this experiment needs to be repeated as a function of phosphorylation of the RLC. There are no other data showing a large reduction in basal ATPase with purified skeletal or cardiac myosin in a folded-back configuration. It is very important, therefore, to demonstrate biochemically with purified human β-cardiac myosin (or HMM) whether de-phosphorylated myosin alone results in a decrease of basal ATPase to the levels seen in the SRX in fibers. If not, then the question is which proteins need to be added (MyBP-C, titin, others) to the myosin to reduce the ATPase activity to the low SRX values. A recent study exploring the impact of homozygous and heterozygous MyBP-C knockout mice on the population of the SRX state revealed that the homozygous knockout of MyBP-C significantly decreased the proportion of myosin heads in the SRX state, as compared to the WT (McNamara et al. 2016), implicating MyBP-C in SRX in vertebrate striated muscle. However, the SRX state has been demonstrated biochemically in tarantula skeletal muscle (Naber et al. 2011), which does not contain MyBP-C. Thus, MyBP-C per se is not necessary for the SRX state in all systems.

## A primary effect of HCM mutations may be to increase N_a_ by reducing the levels of the IHM state in cardiac muscle

Several putative HCM mutations have been mapped on the tarantula IHM, and these cardiomyopathy mutations map to regions that could potentially alter the IHM structure (Alamo et al. 2008; Blankenfeldt et al. 2006; Moore et al. 2012; Waldmuller et al. 2011). The first of such mappings was done on proximal S2 by Blankenfeldt et al. (2006) using the tarantula IHM structure of Woodhead et al. (2005). These authors showed that the HCM mutations R869C or R870H probably affect the structural integrity of proximal S2 by disrupting side-chain hydrogen-bonding networks, while several others lead to charge inversion and cluster within the second negative charge belt (Ring 2) centered around residue E927. Comparison of the structures of WT- and E924K-proximal S2 demonstrates that these mutations are unlikely to have a structural effect (Blankenfeldt et al. 2006). Instead, the HCM mutation E924K has been shown to lead to a loss of binding of the C1–C2 fragment of MyBP-C (Gruen and Gautel 1999), implicating the second negative charge belt in S2 (Ring 2) as the interaction partner of C1–C2. Gruen and Gautel (1999) pointed out that the charge-inverting HCM mutations E927K, E930K and E935K could have a similar effect and that electrostatic interactions may play a role in the regulatory function of cardiac MyBP-C. Subsequently, Alamo et al. (2008) mapped five HCM residues on the tarantula IHM structure and suggested that the close proximity and possible interaction of four proximal S2 mutations (E924K, E927K, E930K, and E935K) with the myosin loop containing R403Q (called the ‘cardiomyopathy loop’) may be helpful in understanding how mutations in the rod region of human cardiac myosin lead to disease. This assignment was corrected, however, in a later IHM model by the same group (Alamo et al. 2016), who then suggested that it is myosin loop 2, not the cardiomyopathy loop, that is responsible for the electrostatic docking interaction with proximal S2. The mapping of seven putative HCM mutations on the motor domain to the tarantula IHM was also reported by Waldmuller et al. (2011), who concluded that the mutations they studied do not correspond to distinct myosin motor domains and suggested a random distribution of mutations in the molecule. However, it is now clear that HCM mutations fall in hotspots, as described above. Finally, Moore et al. (2012) mapped five potentially interacting residues to a tarantula IHM structure and pointed out that HCM mutations lie near these interacting sites; these authors suggested that these cardiomyopathy mutations could potentially alter the interacting head motif. One cautionary note here is that one must be careful in describing which putative HCM mutations are clearly causative of HCM because in many cases HCM mutations are reported from a single individual or from an insufficient number of individuals to be certain that the ‘HCM mutation’ is not simply a normal variant in the population. The inclusion of possible benign ‘HCM mutations’ in structural analyses can easily mislead the spatial analysis and potential clustering of disease-causing HCM mutations on the molecule.

To tie together the mesa hypothesis with the known IHM structure, we asked the question of just where on the IHM structure are the mesa surfaces of the S1 heads? To explore this, we performed homology modeling of three IHM versions of human β-cardiac myosin using the tarantula IHM structures of Alamo et al. (2008) as templates. Two versions (MS01, MS02) (Nag et al. 2017) used Protein Data Bank (PDB) 3DTP (Alamo et al. 2008) as the template and the third (MS03) (Nag et al. 2017) used PDB 3JBH (Alamo et al. 2016) as the template. These were all energy minimized using the YASARA (Krieger et al. 2009) force field. The MS03 model is deposited on Model Archive (http://www.modelarchive.org/project/index/doi/ma-am3yh) and can be downloaded from there. All three of these files can be downloaded from our website (http://spudlab.stanford.edu/homology-models/). The three models are very similar to one another and are all consistent with the conclusions and considerations discussed in our 2016 and 2017 publications (Adhikari et al. 2016; Kawana et al. 2017; Nag et al. 2017). All three models received a good evaluation score by the evaluation metrics TSVMod scores, *Z*-score (DOPE) and GA341 scores as described at https://www.rbvi.ucsf.edu/chimera/docs/UsersGuide/modbase.html. All figures in this review are based on MS03.

When we highlighted the residues of the myosin mesa on our human β-cardiac myosin IHM models, we were amazed to see that the mesas of the blocked and free heads (pink, Fig. 7a) cradle the proximal S2. This observation initiated a series of studies on the physiological regulation of N_a_ as well as the role of N_a_ in the hyper-contractility seen clinically by HCM mutations in both myosin and MyBP-C (Adhikari et al. 2016; Kawana et al. 2017; Nag et al. 2017). Thus, to test the notion that HCM-causing mutations increase N_a_, releasing myosin heads into the actively contracting pool in the absence of adrenergic stimulation, thus leading to the hyper-contractility seen clinically, we examined four HCM-causing mutations that lie in the zone where proximal S2 is thought to bind according to the working model (zone 2; Fig. 7a). Strikingly, each of these four HCM mutations, namely R249Q, H251N and R453C on the mesa and D906G on proximal S2, significantly weakened the affinity of proximal S2 for sS1, the truncated form of S1 missing the RLC, while the three HCM mutations lying outside of zone 2, i.e. R403Q, D239N and R870H, did not (Adhikari et al. 2016; Nag et al. 2017). If the structural model and the overall hypothesis on the effects of HCM mutations on increasing N_a_ are correct, then there should be good correspondence between the localization of HCM mutations and the stability of the various proposed domain interaction sites, and thus far this has proven true. One should, however, design experiments to disprove one’s hypotheses, and there is much experimentation that needs to be done regarding the working hypotheses discussed here.

What other interaction sites are involved in the folded-back IHM off-state? Apart from the interaction between the blocked-head S1 mesa and the proximal S2 tail of myosin, the two heads of the myosin also interact directly with each other (Fig. 7b). These interactions were first reported in chicken smooth muscle HMM 2D crystals (Liu et al. 2003; Wendt et al. 1999, 2001), and then observed in 3D reconstructions from electron micrographs in intact tarantula thick filaments (Woodhead et al. 2005) and analyzed by Alamo et al. (2008, 2016). The interaction between the two heads is mediated by a blocked-head surface, which is adjacent to both the mesa and the actin-binding surfaces, binding to the converter domain of the free head (Figs. 4, 7, bright green residues). Strikingly, most of the known converter HCM mutations lie at this interface, and we hypothesized that they may weaken the head–head interaction, thereby shifting the equilibrium between the closed IHM ‘off state’ and the open ‘on state’ in the direction of increased N_a_ (Kawana et al. 2017; Nag et al. 2017). Appropriate assays need to be developed to specifically probe this S1–S1 interaction, and this is another high priority in the field.

In the sarcomere, other molecular interactions, such as RLC–RLC (Alamo et al. 2008, 2016; Brito et al. 2011; Nogara et al. 2016b), intermolecular head–head (Alamo et al. 2008, 2016; Woodhead et al. 2005), and head–LMM (Alamo et al. 2016; Woodhead et al. 2005) interactions, have been described. In addition, S2–LMM and head–titin interactions may contribute to the IHM state. The interactions of myosin with MyBP-C seem likely to be central to increases in N_a_ by HCM mutations. The C1–C2 interaction with proximal S2 studied by Gruen et al. (Gruen and Gautel 1999) is weakened or abrogated in the disease-causing mutations R870H and E924K. Again, specific assays for focusing on these domain interactions and the effect of local HCM mutations on their affinities need to be established.

The importance of dividing our homology-modeled structure into three zones (Fig. 7a) is to appreciate the location of the HCM-causing mutations at the interfaces of the putative important interactions and to provide a working hypothesis for future experiments. Thus zone 3 mesa mutations, which include R403Q, may weaken the C0–C2-free head interaction (Figs. 6b, 7a), while zone 1 mesa mutations, which include R663H, may weaken the C5–C6-blocked head interactions (Figs. 6c, 7a). Future experiments should be directed at testing such predictions. R403Q is unique in that it lies at the junction of the mesa, the blocked head domain that interacts with the converter surface of the free head (the S1–S1 interaction region), and the actin-binding site for myosin (Fig. 4b). Thus, R403Q could be weakening interactions in zone 3, the S1–S1 interaction region and/or the actin-binding interface.

We emphasize that our models serve the purpose of working hypotheses and should not be viewed as actual structures. As working hypotheses, they can guide experiments to test the models. Another word of caution—we do not consider any of the current IHM models from EM reconstructions to be of sufficient resolution to embark on analyses of the effects of HCM residue changes on neighboring interacting residues, rates of ADP or release of inorganic phosphate, changes in switch 1 and 2, binding affinities between the head and S2 or interactions with MyBP-C, and so forth. Furthermore, the actual human β-cardiac folded IHM structure would be expected to be different in high-resolution detail from, for example, the tarantula skeletal myosin IHM structure, given that more than 40% of residues differ between the S1 heads of these two myosins. High-resolution EM reconstruction images and/or X-ray crystallography structures using purified human β-cardiac myosin or HMM will be necessary to begin to assess the more detailed aspects of the effects of the HCM mutations, and this should be a high priority effort in the field. Nonetheless, our working models of human β-cardiac myosin IHM structures serve as a reasonable guide for experiments to test them. This is one area that is just in its infancy, and a huge amount of work lies ahead.

## Small molecule effectors are promising agents for future treatment of HCM

**Fig. 8:**
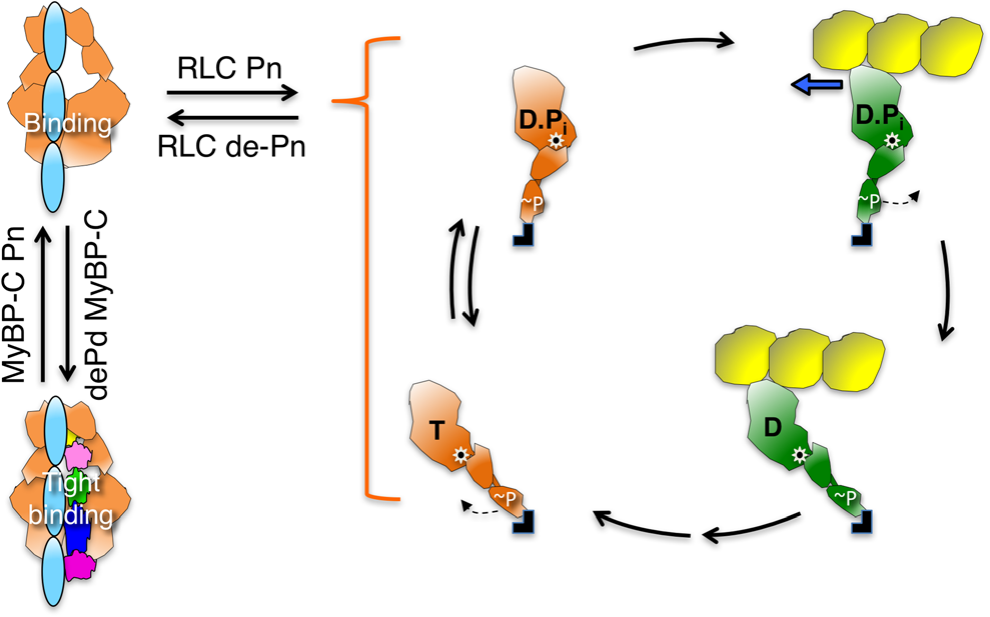
Schematic drawings of the actin–myosin chemomechanical cycle and hypothesized sequestered states of myosin heads. The color schemes of the myosin heads correspond to those in Fig. 1. Steps of the chemomechanical cycle are as shown in Fig. 2. The heads in the cycle are phosphorylated (*~P*) on the RLC. S1 heads (*orange*) that are sequestered into the non-functional IHM state are shown in two states on the *left side* of the figure: RLC de-phosphorylated and bound to their S2 tail (*light cyan*), and complexed with de-phosphorylated MyBP-C, which more firmly locks the heads in the IHM state. Note that other than the interactions shown here, many other interactions, such as those involving LMM and titin, are likely involved in the IHM state of myosin, and a common theme for HCM mutations may be that they shift the equilibrium away from the IHM ‘off state’ of the myosin heads to the ‘open state’ in which the heads are functionally accessible for interaction with actin, thus producing the hyper-contractility observed clinically

There is an urgent need for novel therapeutic interventions for HCM. The current therapeutic approaches aim only to ameliorate symptoms and in severe cases involve open heart surgery to perform myectomies or alcohol ablation of septal coronary arteries—these are not ideal treatments. Effective drug development requires a solid fundamental understanding of the underlying molecular basis for the hyper-contractility observed clinically. While there is much to be learned, molecular understanding of the cardiac contractile system is quite far along. It has been hypothesized, therefore, that a small molecule which binds directly to the very protein carrying the mutation that causes the disease and reduces the hyper-contractility of the contractile system back to normal may obviate the development of downstream effects seen in this disease (Spudich 2014). The South San Francisco-based biotech company MyoKardia (see http://MyoKardia.com) has developed just such an agent, MYK-461, which is a small molecule inhibitor of cardiac contraction (Green et al. 2016) that was first identified from a screen for actin-activated myosin ATPase inhibitors, and thus lengthens the total cycle time (t_c_) of the ATPase cycle. This lengthening results in a reduction of the duty ratio (t_s_/t_c_) and, therefore, of the ensemble force (F_ensemble_), since F_ensemble_ = F_intrinsic_ ⋅ N_a_ ⋅ duty ratio. MYK-461 reduces the contractility of β-cardiac myosin by directly binding to it. This potential drug is in clinical trials and is the first in the line of new HCM therapeutic agents that act directly by interacting with the human β-cardiac myosin and normalizing its power output generating capacity. Given the considerations described in this review, small molecules that specifically shift the equilibrium between the open state of myosin toward the IHM state, and therefore simply lower the amount of N_a_, should be particularly effective drugs for lowering the power output of the system (Fig. 8). The least side effects are likely if one can simply park some heads in their closed ‘off state,’ which avoids altering particular rate constants in the dynamic chemomechanical ATPase cycle. Such a self-limiting system may be an ideal candidate for drug development, possibly with minimal unwanted pleiotropic effects. This approach can also be extended to screening for novel molecules that shift the equilibrium toward the ‘open state’ for treatment of the hypo-contractile disease DCM. A small molecule that has recently been shown to reduce the SRX state levels in fast twitch skeletal fibers, and thus may be destabilizing the IHM state, is piperine (Nogara et al. 2016a). Piperine therefore may be acting to increase N_a_ in skeletal fibers.

## Conclusion and perspectives

Over the past five decades our understanding of muscle physiology, biochemistry and biophysics has greatly advanced. We have a good understanding of how various sarcomeric proteins function and their roles in muscle contractility. Now focus is shifting to understanding the molecular basis of disease states of these proteins. Although a significant knowledge of myosin biomechanics is at hand, there is much to be learned to understand how HCM mutations alter the system at the molecular level to cause the hyper-contractility seen clinically. This is an exciting area of research that deserves a great deal of experimental effort, a perfect area for many new investigators to become involved in. There is much to be done.
